# Large-Scale Functional Networks Identified from Resting-State EEG Using Spatial ICA

**DOI:** 10.1371/journal.pone.0146845

**Published:** 2016-01-19

**Authors:** Stéphane Sockeel, Denis Schwartz, Mélanie Pélégrini-Issac, Habib Benali

**Affiliations:** 1 Sorbonne Universités, UPMC Univ Paris 06, CNRS, INSERM, Laboratoire d’Imagerie Biomédicale (LIB), Paris, France; 2 Sorbonne Universités, Inserm U 1127, CNRS UMR 7225, UPMC Univ Paris 06 UMR S 1127, Institut du Cerveau et de la Moelle épinière, ICM, Paris, France; University of Electronic Science and Technology of China, CHINA

## Abstract

Several methods have been applied to EEG or MEG signals to detect functional networks. In recent works using MEG/EEG and fMRI data, temporal ICA analysis has been used to extract spatial maps of resting-state networks with or without an atlas-based parcellation of the cortex. Since the links between the fMRI signal and the electromagnetic signals are not fully established, and to avoid any bias, we examined whether EEG alone was able to derive the spatial distribution and temporal characteristics of functional networks. To do so, we propose a two-step original method: 1) An individual multi-frequency data analysis including EEG-based source localisation and spatial independent component analysis, which allowed us to characterize the resting-state networks. 2) A group-level analysis involving a hierarchical clustering procedure to identify reproducible large-scale networks across the population. Compared with large-scale resting-state networks obtained with fMRI, the proposed EEG-based analysis revealed smaller independent networks thanks to the high temporal resolution of EEG, hence hierarchical organization of networks. The comparison showed a substantial overlap between EEG and fMRI networks in motor, premotor, sensory, frontal, and parietal areas. However, there were mismatches between EEG-based and fMRI-based networks in temporal areas, presumably resulting from a poor sensitivity of fMRI in these regions or artefacts in the EEG signals. The proposed method opens the way for studying the high temporal dynamics of networks at the source level thanks to the high temporal resolution of EEG. It would then become possible to study detailed measures of the dynamics of connectivity.

## Introduction

The functional organisation of the brain follows two complementary principles known as functional segregation and functional integration [1,2]. The principle of functional segregation asserts that populations of neurons, strongly interconnected (cortical surface area less than 1 cm²), work synchronously and thus form cerebral functional areas clearly delineated spatially, which may be associated with a specific elementary task [3]. For processing a high-level task, several of these functional areas are mobilised and interact at a larger scale with specific temporal dynamics: this is known as functional integration [4]. Functional areas are hence integrated in large-scale functional networks defined as sets of distant cerebral areas that are linked anatomically, through white matter tracts, and functionally, through dynamics of coupled activities [5].

The analysis of these functional networks in healthy subjects [6,7] or in patients [8,9,10] has mainly relied on resting-state functional MRI (fMRI) data. Spatial independent component analysis (sICA) is a widely-used data-driven method that enables reproducible non-invasive mapping of several functional networks [11] at the group level and with a spatial resolution in the order of a few millimetres.

Bayesian inference models (e.g.: dynamic causal modelling [4]) or specific conditional connectivity measures (e.g.: partial correlation [12], conditional integration [13], Granger causality [14]) have been proposed to study the connectivity dynamics within large-scale functional networks in fMRI. However, the frequencies of the natural cognitive rhythms of the brain and the intrinsic dynamics of connectivity in large-scale functional networks remain inaccessible, since fMRI has a limited temporal resolution in the order of the second and hence a frequency spectrum restricted to very low activity frequencies (less than 1 Hz).

These limitations may be overcome by using electroencephalography (EEG) or magnetoencephalography (MEG), which offer a direct measure of neural electrical activity with a temporal resolution in the order of 1 ms.

Several methods have been applied to EEG or MEG signals to detect functional networks. They rely on various approaches such as temporal ICA at the level of the sensors [15], connectivity measures between sensors [16], temporal correlation between fMRI signals and EEG sensors power in several frequency bands [17], or spatial ICA of EEG sensors level and fMRI data for functional network connectivity analysis [18].

Sensor-based approaches suffer from a poor spatial localisation. The interpretation of the resulting maps is compromised by the ambiguity due to the diffusion of the EEG signal. Moreover group studies are difficult, due to variable positions of the sensors leading to different observed physiological processes across subjects [19].

Source localisation approaches give access to the neural correlates of EEG [20,21] with less ambiguity due to volume conduction and thus lead to a greater spatial resolution than sensor-based methods. Frequency tagging has been used by Regan [22], enabling identification of the functional networks involved in the response to a specific stimulus at the cortical level. However, such an activation study (i.e. following the presentation of a specific stimulus) only shows the networks activated by the stimulus and not the other intrinsic functional networks. To identify such networks, resting-state acquisitions (i.e. in which subjects refrain from any overt activity) are preferred.

For instance, in a recent work using MEG and fMRI data, Brookes and colleagues used beamformer spatial filtering combined with temporal ICA of the Hilbert transform of the MEG signals to extract spatial maps of resting-state networks (RSN) [23], which were in accordance with resting-state fMRI networks.

In [24], the authors used dual regression analysis to identify several spatial networks from MEG data, based on seed voxels from independent spatial fMRI networks.

On the other hand, Deliglianni and collaborators [25] analysed simultaneous EEG-fMRI recordings. Following Brookes and colleagues’ work [23], they applied a temporal ICA to the Hilbert transform of the band-limited power of the EEG signals. Then, they used an atlas-based parcellation of the cortex to derive time-series at the cortical level for a further functional connectivity analysis.

Approaches using simultaneous EEG-fMRI recordings offer the opportunity to characterize brain networks with a high spatial resolution based on the fMRI results and a high temporal resolution based on the EEG signals. However, to exploit these simultaneous recordings one needs to fully understand the relationship between both modalities. In that respect, it has been shown that the power of the EEG signal convolved with a canonical hemodynamic response function could be related to the blood oxygen level-dependent (BOLD) signal measured with fMRI [26,27].

Can RSN be spatially characterized in EEG alone at the individual level with a good reliability? Are these networks reproducible at the group level? What is the added value of the high temporal resolution of EEG? And finally, how do the identified networks compare with fMRI findings? In this paper we examine the potential of EEG alone to derive the spatial distribution and the temporal characteristics of large-scale resting-state functional networks.

To do so, we propose an original method that combines an individual data analysis including EEG-based source localisation and spatial independent component analysis, and a group-level analysis involving a hierarchical clustering procedure. This article comprises three sections. Firstly, the method is introduced, which consists of several steps: Source localisation, power computation, individual spatial ICA, group analysis based on hierarchical clustering. Then, to compare the RSN identified from EEG alone with fMRI results, we present the EEG and fMRI protocols and the data acquired in healthy subjects, as well as the comparison methodology.

## Method

Logothetis and Laufs described a link between local field potentials (LFP) and fMRI signal [26, 27, 28]. Their original studies dealt with LFP, thus only rather small populations of neurons were considered (from 10,000 to several millions of pyramidal cells arranged in functional macrocolumns). The associated transfer function was a correlation between the power of the reconstructed cortical sources of the EEG signal convolved with a hemodynamic response function (HRF) and the fMRI BOLD signal [26] (Eq 1):

$$P_{EEG}⊗HRF=X_{fMRI},$$ (1)

where ⊗ denotes convolution, P_*EEG*_ is the power of the reconstructed cortical sources of the EEG signal, X_*fMRI*_ is the BOLD signal recorded in fMRI and *HRF* is the canonical hemodynamic response function used in the framework of the general linear model in conventional fMRI statistical analyses [5]. Persistent coupling between BOLD signal and EEG power has been observed in various frequency bands including Delta and Theta, Alpha, Beta, and Gamma [27].

Given Eq 1 and convolution being linear, one can consider to apply sICA on the power of EEG signals at the source level in several frequency bands to detect functional networks. Thus, we can derive an approach based on Eq 1 consisting of two steps:

1. An individual data analysis involving source localisation and sICA of reconstructed EEG data to identify individual large-scale functional networks and associated temporal dynamics.
2. A group-level analysis based on a hierarchical clustering of individual spatial maps to identify large-scale networks reproducible at the population level.

### Individual data analysis

#### Input Data

Our method required for each subject: the artefact-free EEG signals, the tridimensional positions of the EEG electrodes on the scalp, the meshes of the interface between the grey matter and the white matter (GM-WM interface), and the mesh of the head (surface of the scalp).

#### Data pre processing

**EEG**: Physiological (muscular activity, movement, eye blinks) and non-physiological (50 Hz contamination) artefacts were detected by a variance analysis on the electrooculogram (EOG) and electrocardiogram (ECG) recordings thanks to dataHandler software [29]. The contaminated segments were rejected. These pre processing steps helped to increase the inherently low signal-to-noise ratio (SNR) of the on-going EEG by removing most of the signals not originating from the brain. A better SNR insured an increased accuracy of the source localisation step and a decreased inter-individual variability [30].

**Anatomical MRI**: The brain tissues (grey matter, white matter) were segmented from the anatomical T_1_-weighted MRI of each subject by using the BrainVISA software [31]. A mesh of the GM-WM interface was extracted and decimated to speed up the source reconstruction, while preserving a spatial resolution of the order of 2 mm. Each hemisphere was modelled by a surface of approximately 4,000 vertices, thus a mesh of approximately 8,000 vertices modelled the GM-WM interface for each subject.

#### Localisation of EEG sources

The potential measured in EEG originates from the macrocolumns of pyramidal neurons. These macrocolumns can be modelled as electrical dipoles located at the GM-WM interface and perpendicular to this interface. The decimated mesh of the GM-WM interface was used as the source space comprising approximately 8,000 dipoles per subject. The scalp potentials generated by each dipole depend on the characteristics of the various tissues of the head and are measured by the EEG scalp electrodes. Knowing the geometry of the anatomy and the conductivity of the subject’s head, the time course of the dipoles’ activity can then be assessed by solving two consecutive problems: the forward problem and the inverse problem.

The forward problem consists of modelling the contribution of each dipole to the signals of the EEG electrodes by solving Maxwell’s equations, which take the geometry and conductivity of head tissues into account. In this study, the head was modelled by three nested spheres modelling the different tissues [32]. A relative conductivity coefficient was assigned to each tissue (skin: 1, bone: 1/80, brain: 1). Solving Maxwell's equations yielded a gain matrix G such that

X=G×D, (2)

where the *m* x *t* matrix X represents the signal on *m* EEG electrodes and on *t* time points, and the *n* x *t* matrix D represents the time course of activity for *n* dipoles on *t* time points. G is hence an *m* x *n* matrix, where *m* is the number of electrodes and *n* the number of dipoles, i.e. the number of vertices in the mesh of the GM-WM interface.

The second step for the reconstruction of the signal is the inverse problem. It consists of estimating the signal D at the level of the dipoles from the EEG signal measured on the electrodes, knowing the gain matrix G. To solve this problem, we used the weighted minimum-norm method where the EEG signals were re-referenced using an average reference. The analysis was performed using the BrainStorm software [20].

#### Temporal dynamics

In order to take all the information contained in the EEG data into account and to benefit from their high temporal resolution, five frequency bands of interest were considered, which cover the usual frequency spectrum of EEG data (Delta and Theta 1 to 7 Hz, Alpha 7 to 13 Hz, Beta 17 to 23 Hz, Gamma1 27 to 33 Hz, Gamma2 33 to 40 Hz).

**Fig 1:**
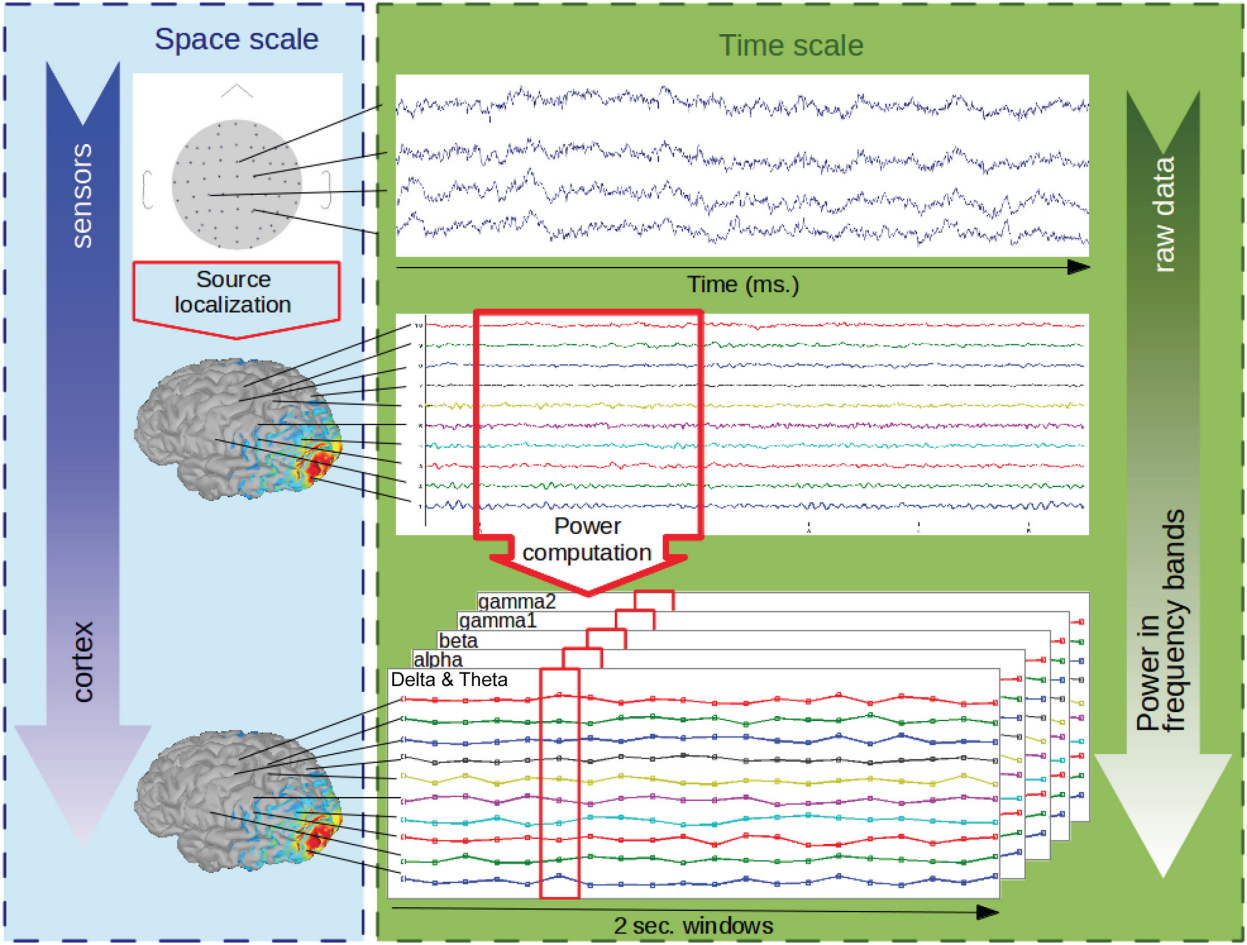
Processing of the EEG data. The artefact-free raw data at the sensors level (top row) were fed into a minimum-norm localisation algorithm to obtain current densities at the cortex level (middle row). After filtering in 5 frequency bands (Delta & Theta, Alpha, Beta, Gamma1, Gamma2), the power of the cortical signals was computed by integrating over two-second-long windows. The end result at the cortical level was the power time courses in each of the 5 frequency bands (bottom row).

Using 2-second-long time windows, the power of the signal was computed at the source level in these 5 frequency bands, using a fast Fourier transform (FFT) on *T* time points. The five *n* x *T* matrices Y_freq_ were concatenated as follows (see Fig 1):

$${\text{Y = [Y}}_{\text{1-7Hz}}{\text{Y}}_{\text{7-13Hz}}{\text{Y}}_{\text{l7-23Hz}}{\text{Y}}_{\text{27-33Hz}}{\text{Y}}_{\text{34-40 Hz}}\text{]}.$$

The power of EEG data was not distributed uniformly over the frequency spectrum. The power in low frequencies was higher than that in high frequencies (1/f effect). In order to take the five frequency bands of interest into equal account, we normalized the data. To reduce the 1/f effect, we computed the logarithm of the ratio of the power to the average power in time and space for each frequency band.

#### Spatial ICA

**Fig 2:**
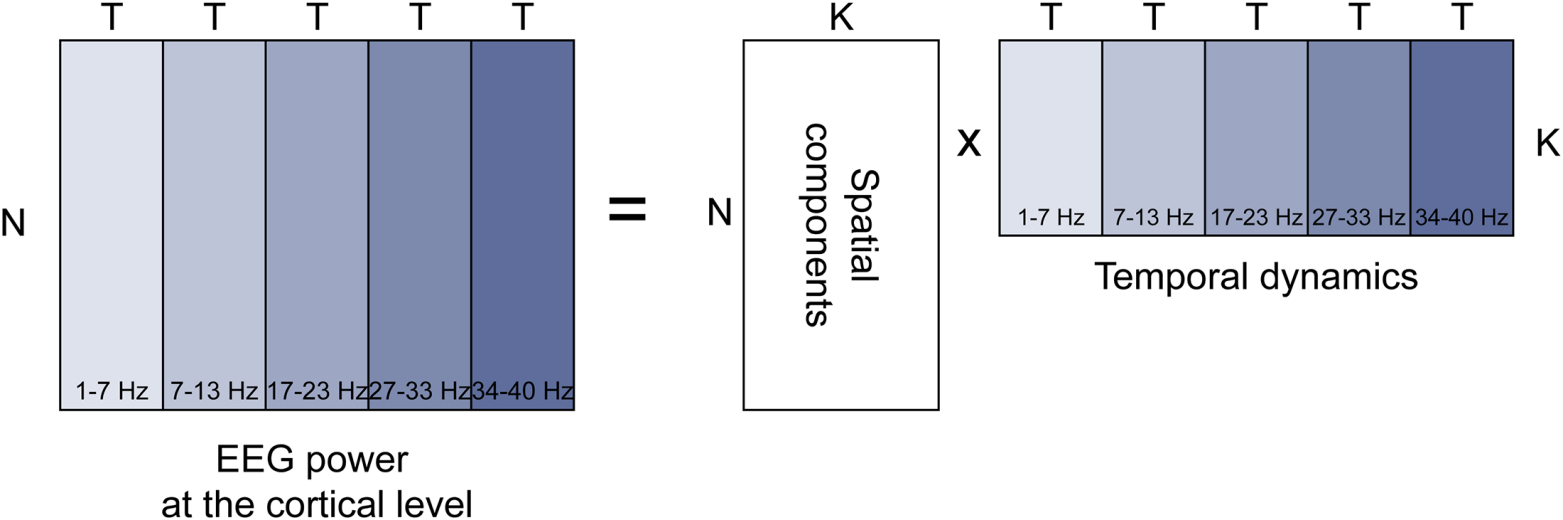
Spatial ICA of the concatenated power dynamics at the cortical level.

In fMRI, methods based on sICA have become the reference to detect large-scale functional networks. Following Eq (1), we applied sICA on the power of the signals associated with the EEG dipoles (Y matrix, see Fig 2). We resorted to the FastICA algorithm [33]. A principal component analysis (PCA) of Y^T^ was first performed in the temporal dimension, followed by an ICA on the spatial dimension of the main PCA factors (i.e. explaining 95% of the total variance). The number of ICA components to retain was set to *K* = 100 for each subject, so as not to neglect any relevant information. Each sICA spatial component corresponded to a network on the GM-WM interface. The associated temporal dynamics corresponded to the time-frequency signature in the five frequency bands of the network.

### Group-level data analysis

Up to now, our approach had remained strictly individual since sICA was performed at the subject level. One hundred components were obtained for each subject, each component representing a spatial distribution on the GM-WM interface of the subject associated with five time courses corresponding to each frequency band of interest. The following group-level analysis was aimed at identifying large-scale networks reproducible at the population level. The group-level analysis reduces the variability observed at the individual level due to the low SNR of on-going EEG.

#### Spatial Normalization of the data

This group analysis requires a common cortical surface to be able to compare the spatial distributions of the components originating from the different subjects. We selected the GM-WM interface colin27 from the Montreal Neurological Institute (MNI), adopted as a standard by the ICBM [34]. The spatial distributions of the individual sICA components were projected to this surface by using Shepard’s interpolation [35].

#### Hierarchical clustering

To identify networks at the population level from the whole set of normalized individual spatial components, the components were clustered according to their spatial similarity using Ward’s criterion [36]. To do so, a dendrogram was built based on the Euclidean distance between the normalised individual components, and this hierarchy was threshold using two criteria [37]. On the one hand, a representativity criterion was defined as the percentage of the total number of subjects contributing to one cluster of the hierarchy. On the other hand, a unicity criterion was defined as the percentage of the total number of subjects contributing with only one component to one cluster of the hierarchy. The assumption was that, for a network to be reproducible across the group, a relevant component would be found in a spatially analogous manner in most subjects with a high representativity and a high unicity.

At the group level we then defined a large-scale network as the spatial average of its individual clustered sICA components in space and frequency bands powers. In order to identify the main cortical areas of this network, it is necessary to develop a statistical model. Following the assumption that the individual sICA components in a given cluster are drawn from an independent random sample, the spatial average of these components follows a Student spatial distribution [37] that can be threshold (p< 0.05), which ensures that the remaining cortical regions are robust across subjects since they are above the chance level.

### Comparison of EEG and fMRI large-scale networks: EEG and fMRI data acquisitions

All data were acquired in the NeuroImaging Research centre (CENIR) at the Pitié-Salpêtrière hospital in Paris, France. This study was specifically validated by the local ethics committee: Comité de Protection des Personnes–Ile-de-France under the number CPP DGS2007-0555. Fifteen right-handed healthy subjects (6 females, aged 26.6 ± 2.1 years) participated in the study after giving their written informed consent.

Two EEG datasets were acquired, one outside the MRI magnet and the other simultaneously with the fMRI acquisitions. Only the EEG data recorded outside the magnet were considered in this study. The subjects were placed under the same conditions as in the magnet: they were lying supine, looking at the stimuli via a mirror and could hear an audio recording equivalent to the noise produced by the MRI gradients. The EEG acquisitions were carried out with a 62-channel BrainAmp cap, associated with an electrocardiogram (ECG) and an electrooculogram (EOG). The sampling frequency of the signal was 5 kHz. The high sampling rate of our EEG signals insured a good stability of the power spectra computed using FFT at the cortical level. We did not notice any instability. The reference electrode was located on the cap on Cz, and the ground electrode below Oz. Electrode impedance did not exceed 10kOhm. The electrodes were placed on the subject’s scalp according to the 10/20 system and their tridimensional coordinates were measured thanks to a Polhemus localisation system. The coordinates of three fiducial points (nasion, left and right pre-auricular) were also recorded.

A preliminary visual inspection of the EEG data enabled us to check their overall quality. Three subjects were excluded, since either the quality of their EEG signal was poor (2 subjects), or the EEG cap was positioned incorrectly (1 subject). Thus, 12 subjects were finally included in the analysis.

The functional and anatomical MRI data were acquired on a Siemens 3 Teslas Trio TIM system (functional data: EPI sequence, TR = 2s, TE = 27ms, flip angle = 78°, matrix size 64x64 voxels, 40 contiguous slices, voxel size: 3 mm isotropic, 750 volumes; T_1_-weighted anatomical data: MPRAGE sequence, TR = 2.3s, TE = 4.18ms, flip angle = 9°, matrix size 256x256 voxels, 176 contiguous slices, voxel size: 1 mm isotropic).

The acquisition protocol included resting-state periods (5 minutes eyes closed), visual and motor tasks. In this paper, only the last resting-state period at then end of the experiment was analysed.

### Comparison strategy

To investigate whether the large-scale functional networks identified in EEG at the group level were similar to those found in fMRI, a comparison methodology was designed. Functional networks were identified from fMRI data using the NetBrainWork software as described by Perlbarg and colleagues [38], which yielded 3D images of fMRI-based functional networks (RSN_fMRI) in the MNI standard space. In order to compare spatially these networks with the functional networks obtained from the EEG data (RSN_EEG) on the surface of the GM-WM interface in the same MNI standard space, the RSN_fMRI were interpolated on the surface of the MNI GM-WM interface, thanks to a method using a Voronoi diagram [39]. We noticed that the RSN_EEG had a much smaller spatial extent than the RSN_fMRI (see the results and the discussion sections). We thus tested whether there existed a linear combination of the RSN_EEG that would correspond to a given RSN_fMRI. To this end, a stepwise regression analysis was applied between RSN_fMRI and RSN_EEG as covariates. By proceeding stepwise, i.e. by adding the RSN_EEG one by one in the regression, only those networks that showed the greatest correlation (tested with a Fisher’s F-test) with each RSN_fMRI were selected.

## Results

### Individual data analysis

Preprocessed EEG signals were first of all analysed individually. The minimum-norm method was used to estimate the activity of sources on the GM-WM interface and this activity was integrated in 2-second-long time windows. Time windows containing an artefact were excluded, thus the number of time windows differed between subjects (119 ± 14.4 windows/subject). Signal powers were then extracted in the five frequency bands of interest.

**Fig 3:**
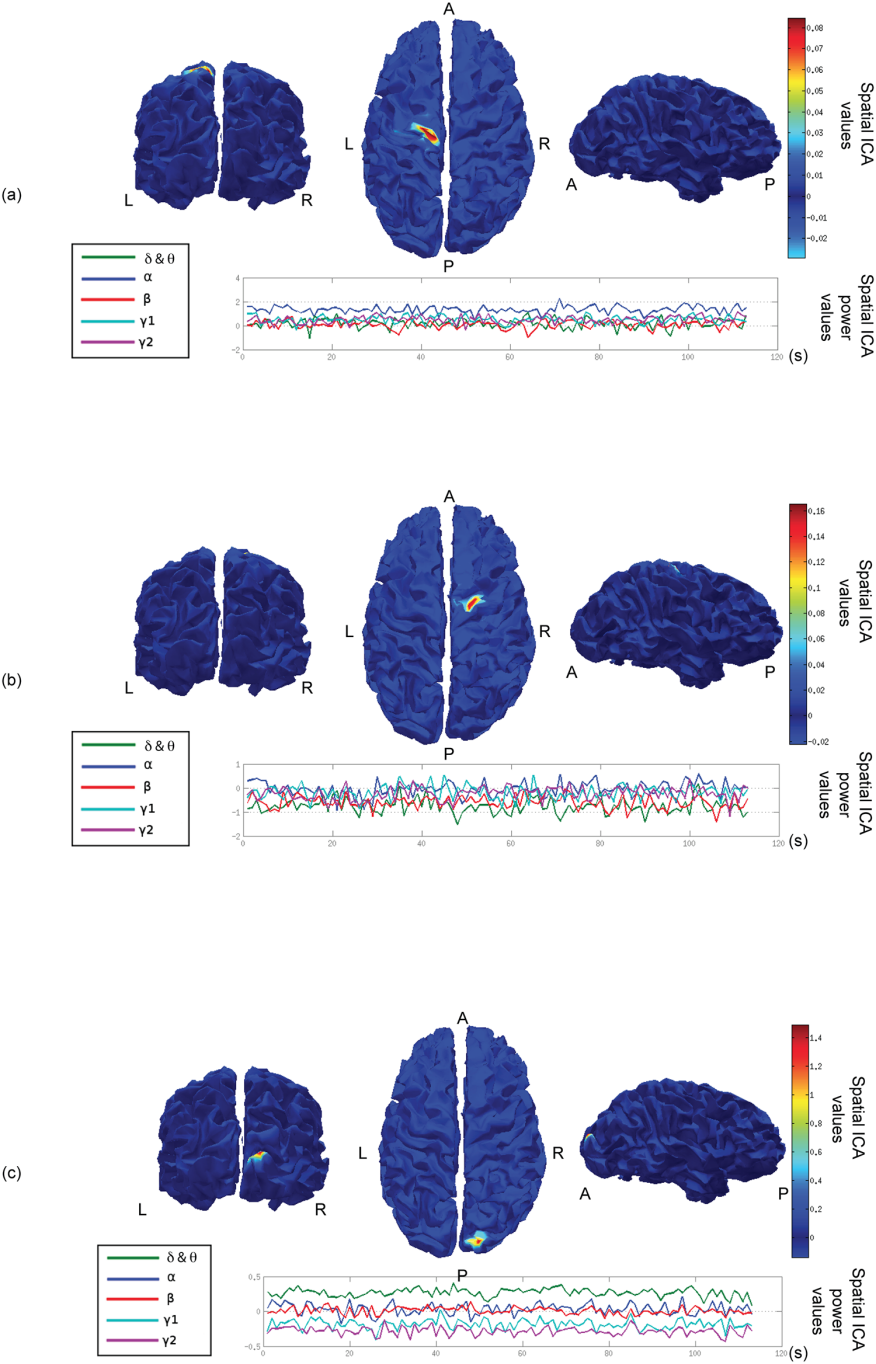
Three individual spatial ICA components. For each component, the top part of the figure shows the values of the spatial distribution. The bottom part of the figure shows the power values of the corresponding component along time for the 5 frequency bands Delta & Theta, Alpha, Beta, Gamma1 and Gamma2.

Spatial ICA was conducted on the matrix Y corresponding to the signal power in the five frequency bands at the source level on the GM-WM interface. For every subject, the first 100 components were considered. Three of these components are shown in Fig 3 for one subject. Every component had a spatial distribution (top rows of Fig 3a, 3b and 3c) and 5 time-frequency representations corresponding to the dynamics of power in the 5 frequency bands of interest (bottom rows of Fig 3a, 3b and 3c).

All the spatial components had a small extent (approx. 50 vertices), showing one main area with high values of the spatial distribution and many other areas associated to lower values, distributed more sparsely over the whole GM-WM interface. The 100 highest values areas covered the whole GM-WM interface,

One of the major interests of the proposed method is that it allowed us to study the time-frequency distribution of each spatial component in specific bands of interest.

For each component an equivalent contribution of the power in the five frequency bands was observed (see Fig 3b). However, some of the spatial components contained more power in one frequency band, see Fig 3 for the Alpha (3a) band, the Delta and Theta band (3c).

### Group-level data analysis

**Fig 4:**
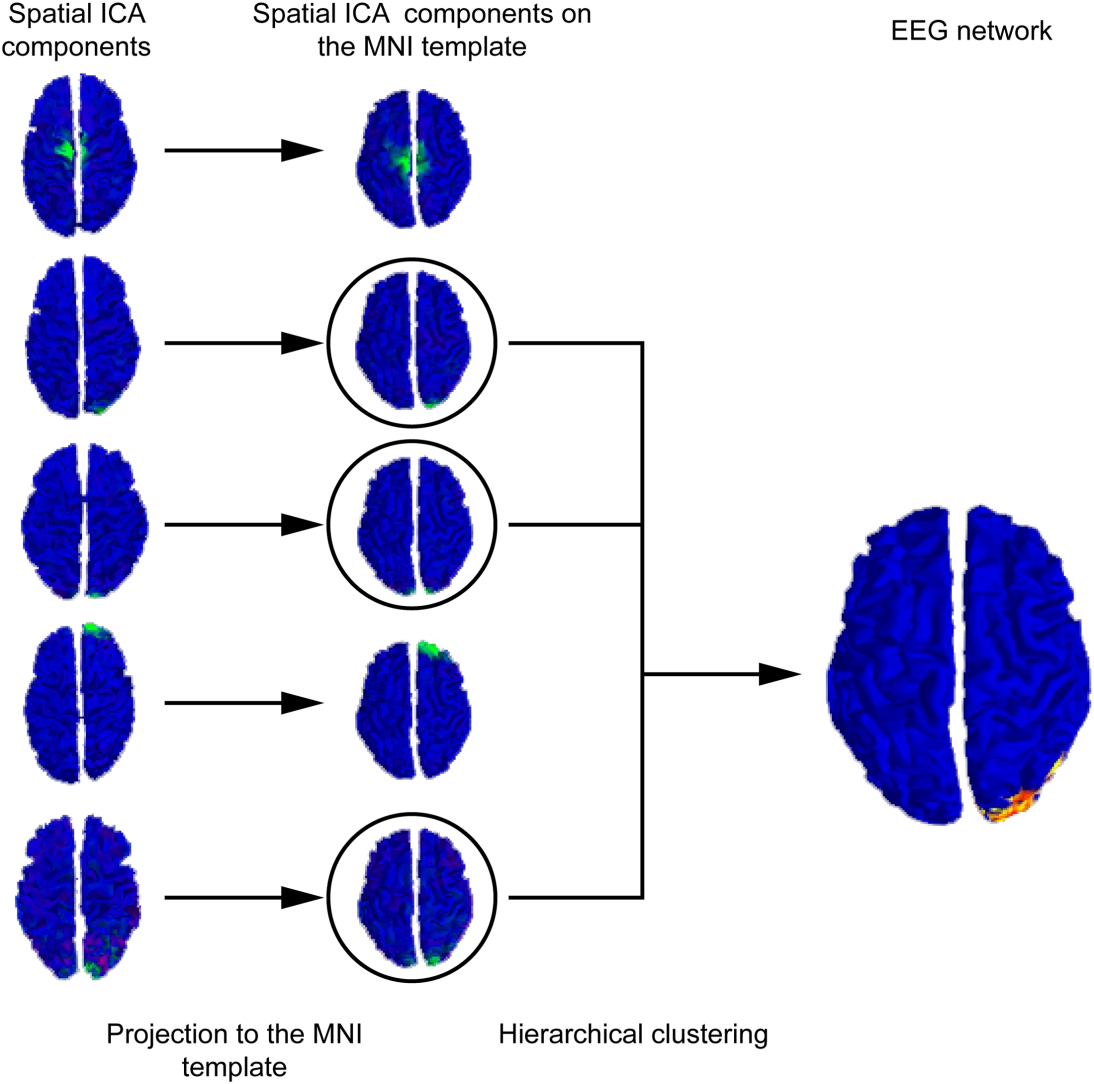
Hierarchical clustering of the individual spatial ICA components. The individual spatial ICA components (left) were projected to the MNI colin27 template (right). A hierarchical clustering was then performed to identify the RSN_EEG.

Once one hundred components were extracted for each subject, they were projected onto the standard MNI colin27 surface. Hierarchical clustering among all the individual sICA components was then performed. Fig 4 shows examples of clusters obtained using a representativity criterion superior or equal to 0.5 and a unicity criterion superior or equal to 0.3. This hierarchical clustering yielded to 104 clusters. 92 out of the 1200 components obtained from the individual sICAs could not be clustered in any cluster according to the representativity and unicity criteria (See Fig 4). The spatial average map in each cluster was then computed and threshold using t-statistics (see section “Hierarchical clustering”) at p<0.05 corrected for multiple comparisons using a false discovery rate procedure.

**Fig 5:**
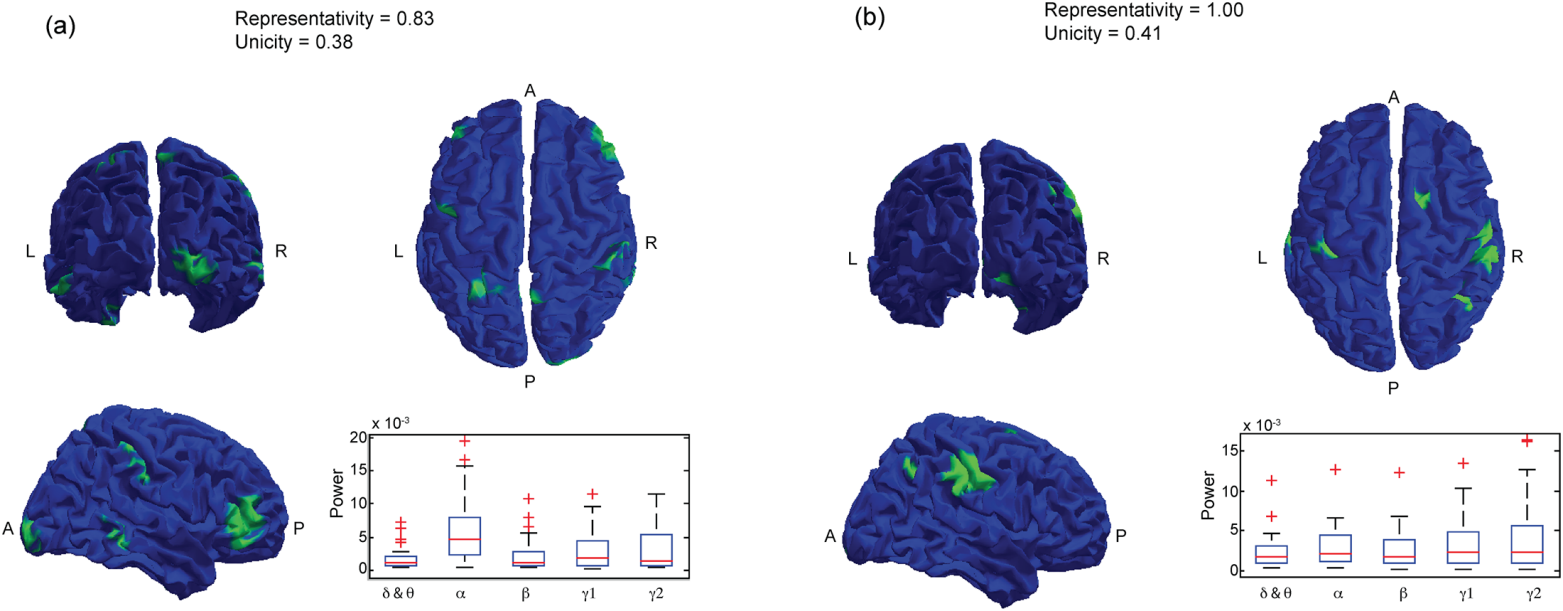
Two examples of RSN_EEG. Two RSN_EEG and associated power distribution in each frequency band are shown. (a) RSN_EEG including somato-motor areas and (b) RSN_EEG including fronto-parietal areas.

The final 104 RSN_EEG contained between 10 and 38 individual sICA components from several subjects. Fig 5 shows two RSN_EEG and associated power distribution in each frequency band. Fig 5a shows an RSN_EEG including somato-motor areas. All subjects contributed to this network (representativity = 1), 41% with only one component (unicity = 0.41). Power distribution was equally distributed over the five frequency bands. The estimated variance was lowest for the low-frequency bands. Fig 5b shows an RSN_EEG including fronto-parietal areas. 83% of the subjects contributed to this network (representativity = 0.83), 38% with only one component (unicity = 0.38). Power distribution was equally distributed except for the Alpha band.

### Comparison of EEG and fMRI large-scale networks

Twenty functional networks were extracted at the group level from fMRI data and projected onto the MNI standard GM-WM interface. The extracted RSN_fMRI were those conventionally identified including a motor / sensori-motor network, a visual network, and the default mode network.

**Fig 6:**
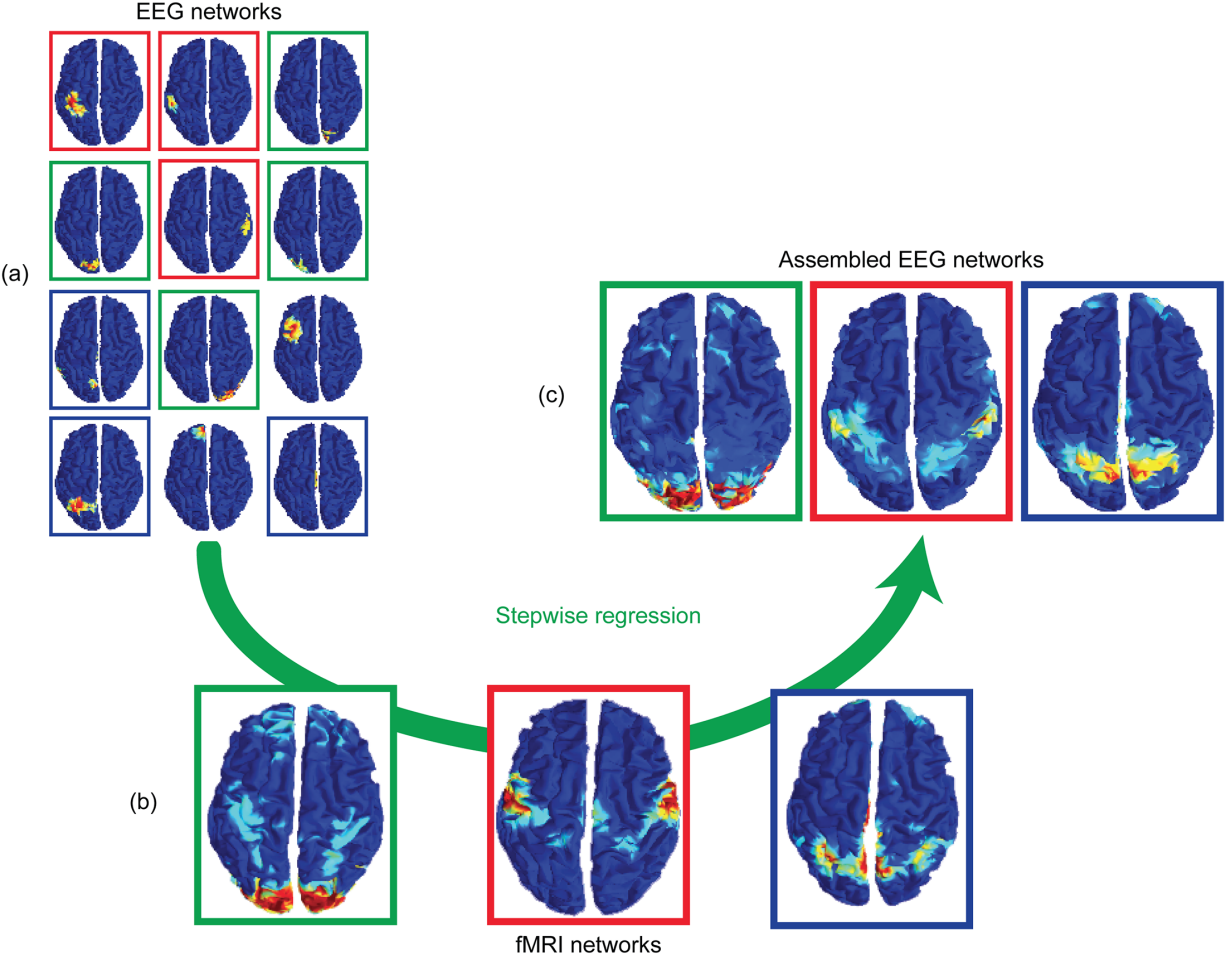
Stepwise regression performed between RSN_EEG and RSN_fMRI. The results of the stepwise regression performed between RSN_EEG (a) and RSN_fMRI (b) are shown in (c).

Fig 6 shows the result of the stepwise regression analysis between EEG and fMRI networks. A linear combination of few EEG networks was found to overlap closely each of the fMRI networks. Fig 6 illustrates such an overlap for the visual, motor and default mode networks, respectively.

**Fig 7:**
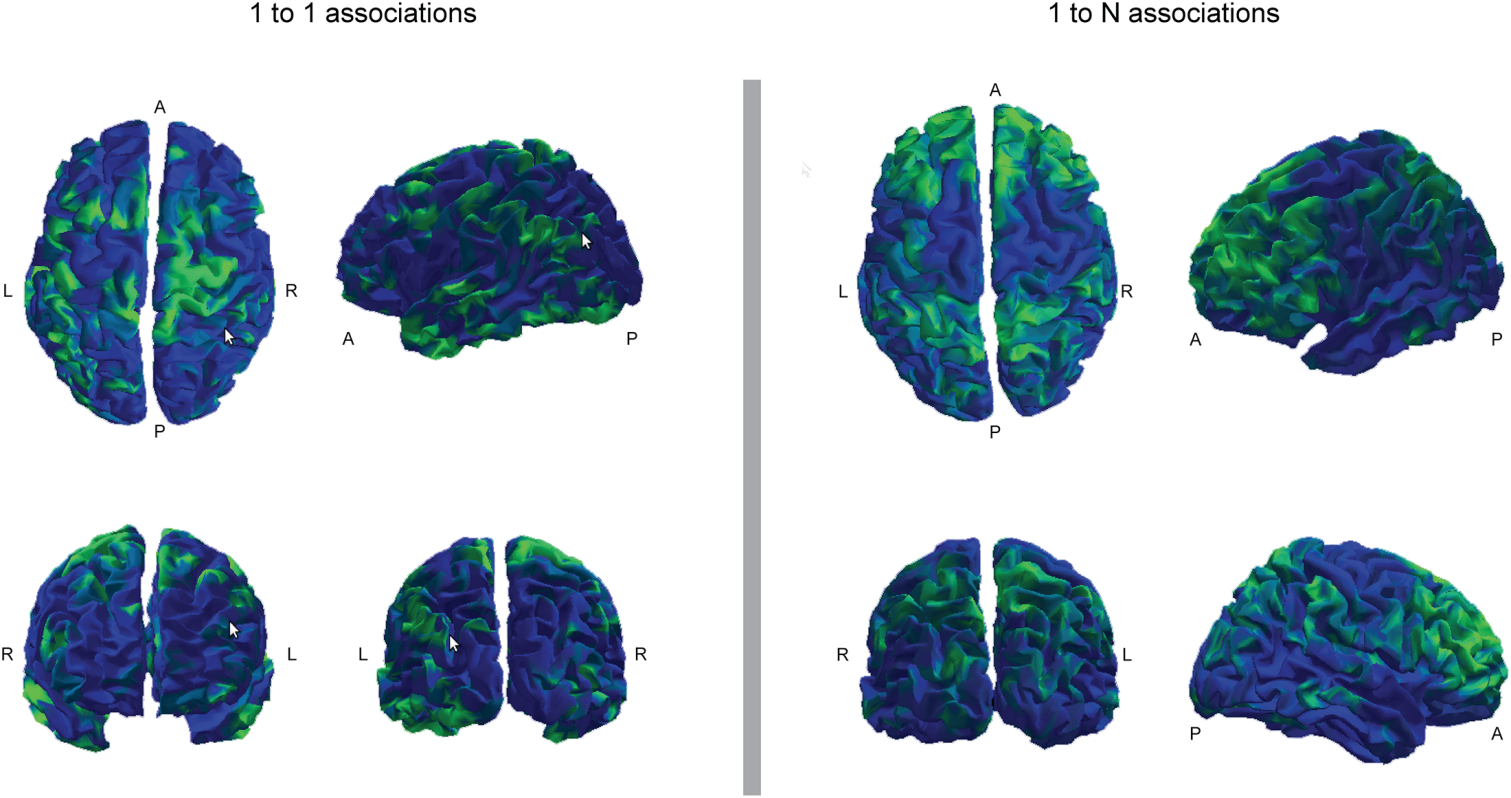
Association of EEG networks with fMRI networks. The RSN_EEG overlapping with only one RSN_fMRI are shown in the left part of the figure (1 to 1 association). The RSN_EEG overlapping with several RSN_fMRI are shown in the right part of the figure (1 to N association).

**Fig 8:**
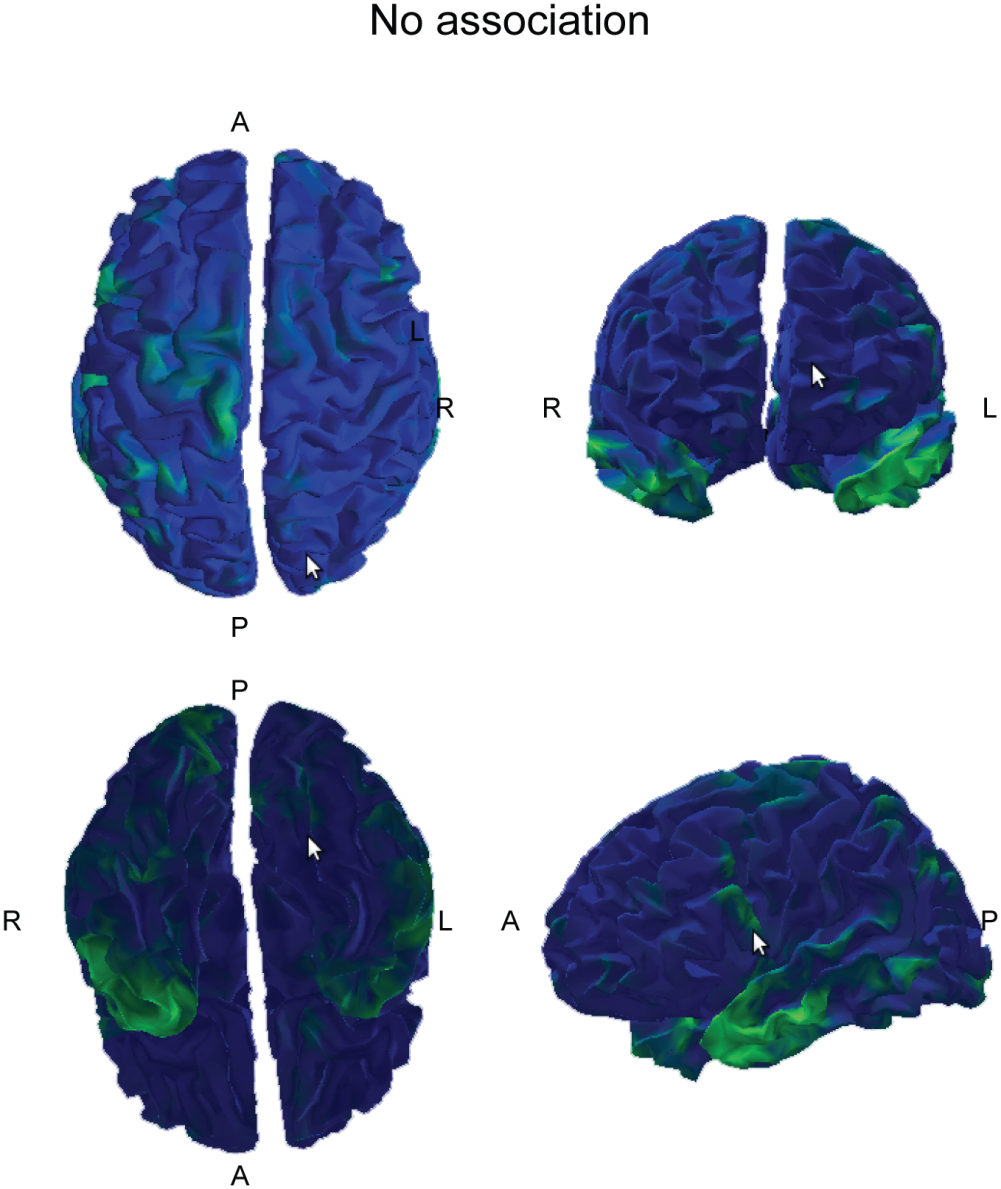
EEG networks not included in any fMRI network.

Among the 104 RSN_EEG, some overlapped with only one RSN_fMRI, others with several RSN_fMRI. The left part of Fig 7 shows the 23 RSN_EEG that overlapped with only one RSN_fMRI (1 to 1 association). The 47 RSN_EEG overlapping with several RSN_fMRI are shown in the right part of Fig 7 (1 to N association). Finally the 34 RSN_EEG not overlapping with any RSN_fMRI are shown in Fig 8.

Association of one EEG network with one fMRI network only was found mainly in motor, premotor and sensory areas. Association of one EEG network with several fMRI networks was found mainly in frontal and parietal areas. EEG networks associated with no fMRI network were found mainly in the anterior temporal lobes.

These results showed that large-scale functional networks could be characterized using EEG alone with a good reliability.

## Discussion

### Discussion of the method

We proposed an approach to characterise spatially and temporally brain networks using EEG recordings while taking into account information in several frequency bands. The method consisted of two steps:

1. An individual data analysis including EEG-based source localisation and spatial independent component analysis
2. A group-level analysis including a hierarchical clustering of spatial individual components.

At the individual level, the proposed multi-frequency data analysis allowed us to characterize the networks using a source localisation approach (forward / inverse problem) and a spatial ICA. At the group level, we used hierarchical clustering to identify which large-scale networks were reproducible across the population. Besides, we used a regression analysis to compare EEG and fMRI networks. At each step, we used validated methods, commonly used in other contexts; however each of them had its limitations.

We used the concentric sphere method to solve the forward problem [32]. It is a simple technique of low computational cost. However, this method is less realistic compared with approaches such as the boundary element method, which may allow obtaining a better spatial resolution by using the individual anatomy in the model. Focusing on the cortex, we did not take into account in our model deep brain structures such as the thalamus and the hippocampus, which play an important role in processing high-level information. New models including deep structures are now available [40]. Their use would enable a full characterisation of brain networks with EEG. However, since subcortical regions have not been observed in a reproducible manner with fMRI, this would make a comparison between the two modalities difficult.

We used minimum-norm estimation to solve the inverse problem. Other localisation approaches could be used in our framework such as LORETA [21] or beamformers [23], which may improve the frequency and spatial characterisation of the networks. It had been shown that the choice of the reference and the volume conduction influence the recorded brain activity. In our case, the optimization of the re-referencing of the EEG by using an infinity reference as proposed by Qin and collaborators [41, 42] with REST algorithm would improve the reconstruction at the source level and further computation of functional connectivity.

Reconstructing sources at the cortical level allows disambiguating the EEG surface signals up to a certain level. However, due to the inverse problem being ill-posed, the spatial resolution of EEG remains low (in the order of centimetre). In our method, we did not use directly a connectivity measure to identify the networks at the individual level. Our results did not show a strong leakage effect. Indeed, sICA, by using both space and time, allowed a good separation of the networks by finding a set of cortical regions that followed a similar temporal behaviour (See Fig 3). The results at the group level showed a lower spatial resolution due to inter-individual variability. The spatial resolution of our approach could be further improved by the re-reference through the REST algorithm and the use of optimization approaches as described in recent papers [43] leading to a strong decrease in spatial leakage at the source level.

A significant tuning parameter for sICA was the number of independent components extracted for each subject. The number of components was set to *K* = 100 for each subject, similar results were obtained using *K* = 200. We believe that K = 100 should be adequate for resting-state studies on normal subjects. This parameter may be lowered when analysing signals obtained while performing a task or filtered signals in a single frequency band.

At the group level, we defined a RSN_EEG as the spatial average of its individual clustered sICA components in space and frequency bands powers. To determine the RSN_EEG of interest, we used two thresholds: representativity and unicity adapted from the fMRI literature (0.5 for representativity and 0.3 for unicity), we obtained 104 classes with a small spatial extent. The chosen unicity parameter was lower than the one used in fMRI [38]. This is probably due to the small spatial extent and the higher number of the individual sICA components in EEG compared with fMRI. The representativity criterion quantified the reproducibility of the network at the individual level, a representativity of 0.5 meaning that only half the subjects showed this network. This inter-individual variability observed at the cortical level could be related to the low SNR of the on-going EEG signals and the intrinsic variability between subjects. Thanks to the group-level analysis (hierarchical clustering followed by thresholding of the average map in each cluster), large-scale networks reproducible across the population could be accurately identified.

Another way to perform the group level analysis would involve an ICA on the concatenated Y matrix of all the subjects as proposed in the fMRI literature [44, 45]. In principle this approach could take advantage of the number of subjects to increase the information available to the spatial ICA. If the powers of the sources at the cortical level in the different frequency bands were homogenous across the population of subjects, the results would be similar to those obtained with our method. However, as we mentioned, the signals at the cortical level showed inter-individual variability in space and power. This variability would bias a spatial ICA applied directly at the group level. Indeed, the first independent components could be related to only few subjects with high powers and homogenous spatial distributions. Performing the spatial ICA subject by subject insured that the intrinsic variability of each subject was taken into account in our framework. However this latter approach could be less sensitive to extract not very well integrated networks.

In recent works using MEG and fMRI data [23,24] or EEG and fMRI [25], beamformer spatial filtering combined with temporal ICA analysis was used to extract spatial maps of resting-state networks with [25] or without [23] an atlas-based parcellation of the cortex. Temporal ICA was applied on the concatenated dataset across subjects and for each of the 5 frequency bands (1Hz to 40Hz). In contrast with our method, the extracted networks corresponded to one frequency band.

Since the links between the fMRI signal and the electromagnetic signals are not fully established, and to avoid any bias, we examined whether EEG alone was able to derive the spatial distribution and temporal characteristics of functional networks. Using EEG data alone we obtained resting-state networks the spatial extent of which was smaller than that of the networks found in fMRI on the same population. Linear combinations of RSN_EEG overlapped significantly the RSN_fMRI, as shown by the stepwise regression analysis.

We can conclude that the power of the reconstructed EEG signal in the chosen frequency bands included enough spatial structuring information, which allowed us to extract large-scale functional networks from resting-state EEG data. We did not make any assumption concerning the contribution of one frequency band to a given network as cross-frequency coupling was shown to be a preeminent feature of on-going signals [46]. The power distribution was variable over the five frequency bands across RSN_EEG. An equivalent contribution of the power to the five frequency bands was generally observed. However, some networks contained more power in one frequency band. This is consistent with the observed coupling between fMRI BOLD signal and EEG signal in various frequency bands [27]. Our framework could be applied to a single frequency band, however it may fail to detect many networks where complex cross-frequency coupling would occur.

We chose a 2-second-long time window to compute the power of the signal at the cortical level to be consistent with the repetition time of the fMRI acquisition. However, the length of the time window could be adapted to the frequency content of interest in a given study. For example, to investigate slower dynamics in the on-going EEG signals, the length of the time window could be increased.

Our analysis revealed a hierarchical-like organization of RSN_fMRI into smaller independent RSN_EEG. In fact, a given RSN_fMRI was similar to a set of RSN_EEG thanks to the high temporal resolution of EEG. This result is meaningful with respect to the principles of segregation and functional integration [3].

The comparison strategy yielded a good overlap between the EEG and fMRI networks over a large portion of the cortex. However, there were mismatches between EEG-based and fMRI-based networks. The RSN_EEG associated with a single RSN_fMRI network were found mainly in motor, premotor and sensory areas. Other RSN_EEG associated with several RSN_fMRI were found mainly in frontal and parietal areas. In these areas the RSN_fMRI themselves overlap [47], therefore the RSN_EEG networks were naturally included in several fMRI networks. Moreover, the assumption that functional networks are disjoint from one another may not be justified for example in associative areas. Many areas of the brain have several functions and thus can participate in different large-scale networks. The observed mismatch between the remaining RSN_EEG and RSN_fMRI may result from a poor sensitivity of fMRI in these regions (partial volume effects, deformation), artefacts in the EEG signals (residual ocular, muscular or cardiac artefacts) or neuronal activities that did not induce detectable BOLD signals.

The similarities between EEG and fMRI functional networks might be explained by the fact that these networks are likely to be supported by the same underlying anatomy. To assess this relationship between structure and function, anatomical networks obtained by diffusion imaging [48] could be compared with EEG and fMRI functional networks.

## Conclusion

We proposed an approach to characterise spatially and temporally brain networks using EEG recordings. We examined whether EEG alone was able to derive the spatial distribution and temporal characteristics of functional networks. To do so, we proposed a two-step original method: 1) An individual multi-frequency data analysis at the cortical level. 2) A group-level analysis to identify the reproducible large-scale networks. This EEG-based analysis revealed small independent networks thanks to the high temporal resolution of EEG. The comparison with fMRI showed a good overlap between the EEG and fMRI networks in motor, premotor, sensory areas, frontal and parietal areas. However, there were mismatches in temporal areas resulting from a poor sensitivity of fMRI in these regions or low SNR in the EEG signals.

The advantage of the proposed method is the perspective to study the high temporal dynamics of networks at the source level thanks to the high temporal resolution of EEG. It would then become possible to study detailed measures of the dynamics of connectivity in cortico-subcortical networks and between networks (e.g. measures of coherence or causality). This would provide ideal tools for studying normal and pathological brain function.
